# Scientific Data in the Grinding and Burring of Teeth

**Published:** 1920-10

**Authors:** Emile Huet

**Affiliations:** Brussels


### SCIENTIFIC DATA IN THE GRINDING AND BURRING OF TEETH

BY EMILE HUET, BRUSSELS

This method of work depends on the use of powerful electric motors capable of great speed and of being stopped instantaneously. My earlier demonstrations rested on my own personal experience, but the results were so important that I resolved to support them by scientific data. I constructed two pieces of apparatus to enable me to determine the time, speed and pressure necessary to obtain the maximum work with minimum pain from a cutting tool.

I continued my experiments with a small motor having ten thousand revolutions a minute. By its use I succeeded in diminishing the pressure necessary for grinding by one-half; in the case of hardest enamel two hundred grammes pressure only were needed where four hundred had been required previously, or about one-tenth of the pressure required with the old instrument; one may imagine the delicacy and precision now obtainable.

Rapidity of rotation does away with shakiness and heating disappears as a result of the tenuity of the particles removed.

In cutting through a healthy tooth which has not been anesthetized four zones are met, presenting different degrees of sensibility and necessitating a knowledge of special instrumentation to be used for each.

This is important both from the point of view of rapidity of penetration as well as that of the tolerance of the tissues to the instruments selected.

The first zone is that of the enamel which is removed painlessly by thin stones 20 mm. in diameter, revolving 9,000 times a minute, a pressure of 200 grammes being used. This speed is equal to 565 metres a minute, but a stone 2 mms. in diameter is equal to only 72 metres, and causes slight pain; slight heating also arises owing to the increase of pressure, 500 grammes being necessary.

The amelo-dentinal junction is the second zone and most sensitive on account of lack of the most appropriate instruments. The best results are obtained by continuing the grinding until a tiny area of dentine is exposed, when a drill is substituted for a stone. The speed should be 4,000 revolutions a minute, there being a period of interruption of three-fifths of a second for every two-fifths work. Pressure 600 grammes.

The dentine constitutes the third zone; passing through it there is no great pain on account of the ease with which the dentine is removed. Carefully sharpened and prepared drills are necessary and should be cut so as to penetrate at least 2 millimetres a second with the instrument revolving 5,000 times a minute. Pressure 400 grammes. The drills of commerce do not fulfil the necessary conditions: I use old fissure burs cut for the purpose, they cut at different angles according to the density of the tissue on which they are to be used. The odontoblastic zone is the fourth and is reached about 1 mm. from the pulp. This is cut through rapidly and its passage is generally better tolerated than in the case of the second zone.

Under the influence of the violent traumatism, the pulp can be extripated immediately in a painless manner if one operates rapidly.

In the case of carious teeth diagnosis assumes an importance which is not necessary in the case of healthy teeth.

Sensibility of the dentine is intimately bound up with irritation of the canaliculi which are exposed by the caries.

In operating the first layer must be passed through rapidly, after which the sensation becomes normal.

If the pulp is hyper-sensitive, passage of the drill into its superficial layer causes a little pain. In acute pulpitis, cutting of the pulp, which is momentarily painful, causes the pain to cease at once, but in more acute conditions the painful phenomena are only got rid of by the application of caustics and anesthetics. Hypertension of the contents of the canaliculi play a part in the transmission and persistence of painful sensations.—*L'Odontologie,* May 30, 1920.

